# Non-Small Cell Lung Carcinoma Presenting With Severe Tracheal Deviation: A Case Report

**DOI:** 10.7759/cureus.8890

**Published:** 2020-06-28

**Authors:** Oluwatoyin Dada, Steven Douedi, Tiffany Purewal, Mohammad Hossain

**Affiliations:** 1 Internal Medicine, Hackensack Meridian Health, Jersey Shore University Medical Center, Neptune, USA; 2 Internal Medicine, Jersey Shore University Medical Center, Neptune, USA

**Keywords:** non-small cell lung carcinoma, tracheal deviation, lung cancer, adenocarcinoma, malignancy

## Abstract

Non-small cell lung carcinoma (NSCLC) is usually diagnosed in adults between the ages of 18-35 years. Here, we present a case of a young adult who presented with severe tracheal deviation and was diagnosed with advanced-stage NSCLC with no past medical or social histories; she presented with worsening right arm pain, numbness, and tingling over the course of three weeks. She presented to an urgent care center that prescribed her nonsteroidal anti-inflammatory drugs (NSAIDs) and muscle relaxants without any symptom improvement. Her pain continued to worsen, and she went to the emergency department (ED) where an X-ray and CT angiogram were performed revealing a large right lung mass severely compressing the trachea. Biopsies confirmed non-small cell lung cancer and the patient was started on chemotherapy. As clinicians, sometimes we do not pay attention to young patients with nonspecific complaints. This case clearly reflects the importance of proper evaluation of symptoms even though there is low clinical suspicion.

## Introduction

Non-small cell lung carcinoma (NSCLC) makes up about 80% of all lung cancer diagnoses. NSCLC is divided into multiple subtypes, with adenocarcinomas being the most diagnosed. Features of adenocarcinomas include, but are not limited to peripheral masses, subpleural nodules, or malignant pleural effusions. In terms of demographics, females are more affected compared to males. It has been noted that women with adenocarcinoma are often non-smokers, suggesting that other factors are at play, including mutations or hormonal differences [1].

We present a case of a young African-American woman who initially presented with pain and numbness of her right arm without respiratory symptoms and found to have severe tracheal deviation due to stage IV lung adenocarcinoma.

## Case presentation

**Figure 1:**
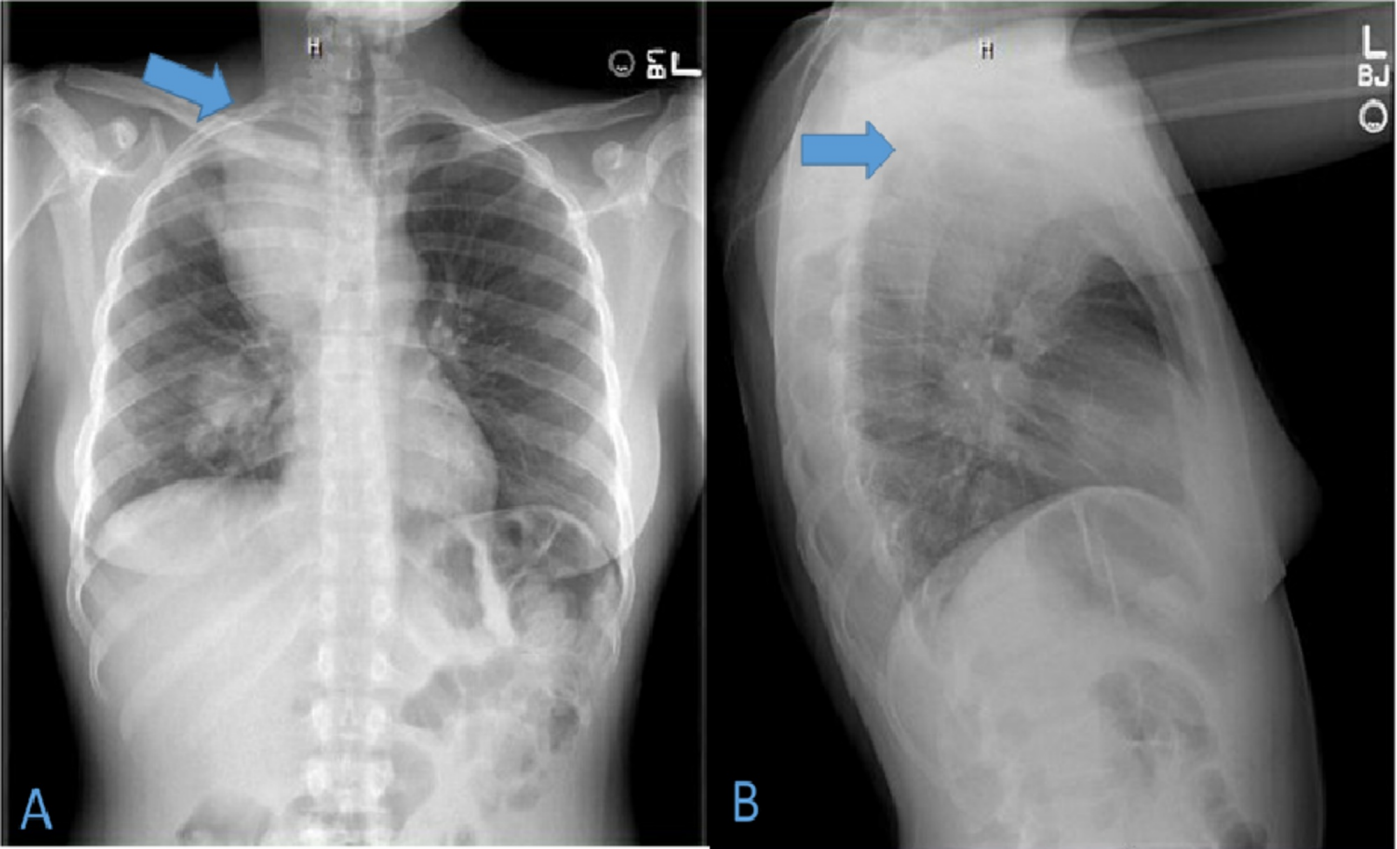
Postero-anterior and lateral view of chest X-ray 1A. Postero-anterior view of the chest showing the right upper lung mass, indicated by the blue arrow. 1B. Lateral view of the chest showing the right upper lung mass, indicated by the blue arrow.

A 24-year-old Haitian female with no known past medical history or smoking history was in her usual state of health until three weeks prior to presentation when she started to experience right arm pain, numbness, and tingling sensation. She did not report a history of cancer in her family. She initially presented to an urgent care facility where she was prescribed nonsteroidal anti-inflammatory drugs (NSAIDs) and muscle relaxants without any improvement of her symptoms. She also had two episodes of pre-syncope over the past two months which she attributed to poor diet; she did not seek any medical attention. On the day of presentation to the emergency department (ED), the patient was returning from the Bahamas via airplane when she felt progressively worsening right-sided chest discomfort radiating to the right flank and back. She reported right upper extremity pain and numbness. She did not report cough, fever, shortness of breath, cold symptoms or recent changes in the weight and appetite. Her vitals in ED were a heart rate of 120 beats per minute, oxygen saturation of 98% on room air, blood pressure of 110/78 mm Hg, and temperature of 98.2 F. Her physical examination was unremarkable. Initial laboratory investigation was normal including complete blood count (CBC) and comprehensive metabolic panel (CMP). A preliminary chest X-ray was done (Figure 1).

**Figure 2:**
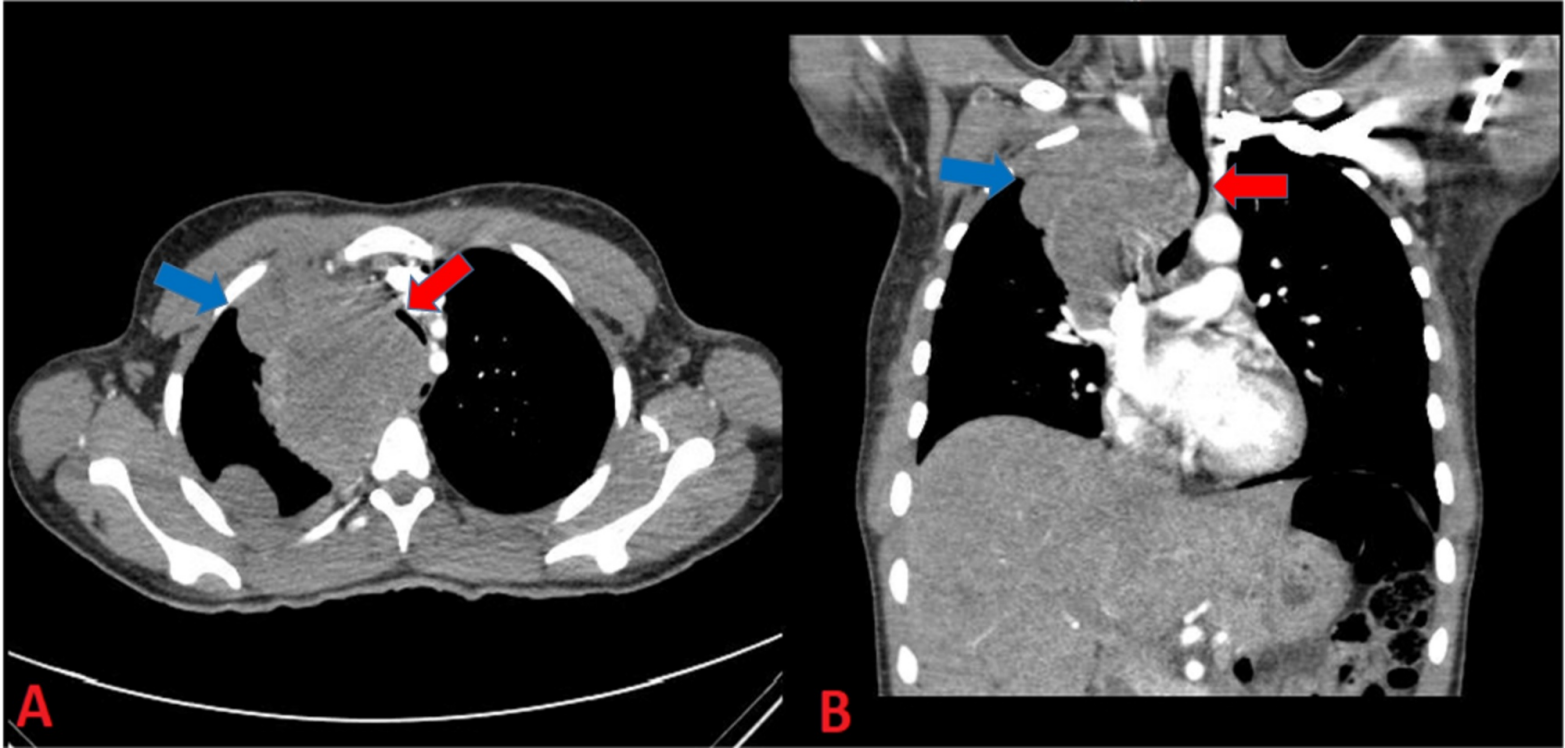
Computed tomography angiography (CTA) of the chest (axial and sagittal view) 1A. CT scan of chest showing a heterogeneous superior right mediastinal mass measuring 10 cm craniocaudally and a 3.6 cm mass in the right middle lobe (blue arrow) and severe tracheal stenosis (red arrow). 1B. CT scan of chest showing lung mass (blue arrow) compression leading to “coin slit” trachea (red arrow).

Due to tachycardia and positive travel history, a computed tomography angiogram (CTA) was performed which was negative for a pulmonary embolism. However, CTA revealed a heterogeneous superior right mediastinal mass, measuring about 10 cm. The trachea was noted to be severely deviated to the left and markedly stenosed. Multiple pleural-based lung masses and nodules were also present on imaging, which are classical imaging signs of adenocarcinoma (Figure 2).

**Figure 3:**
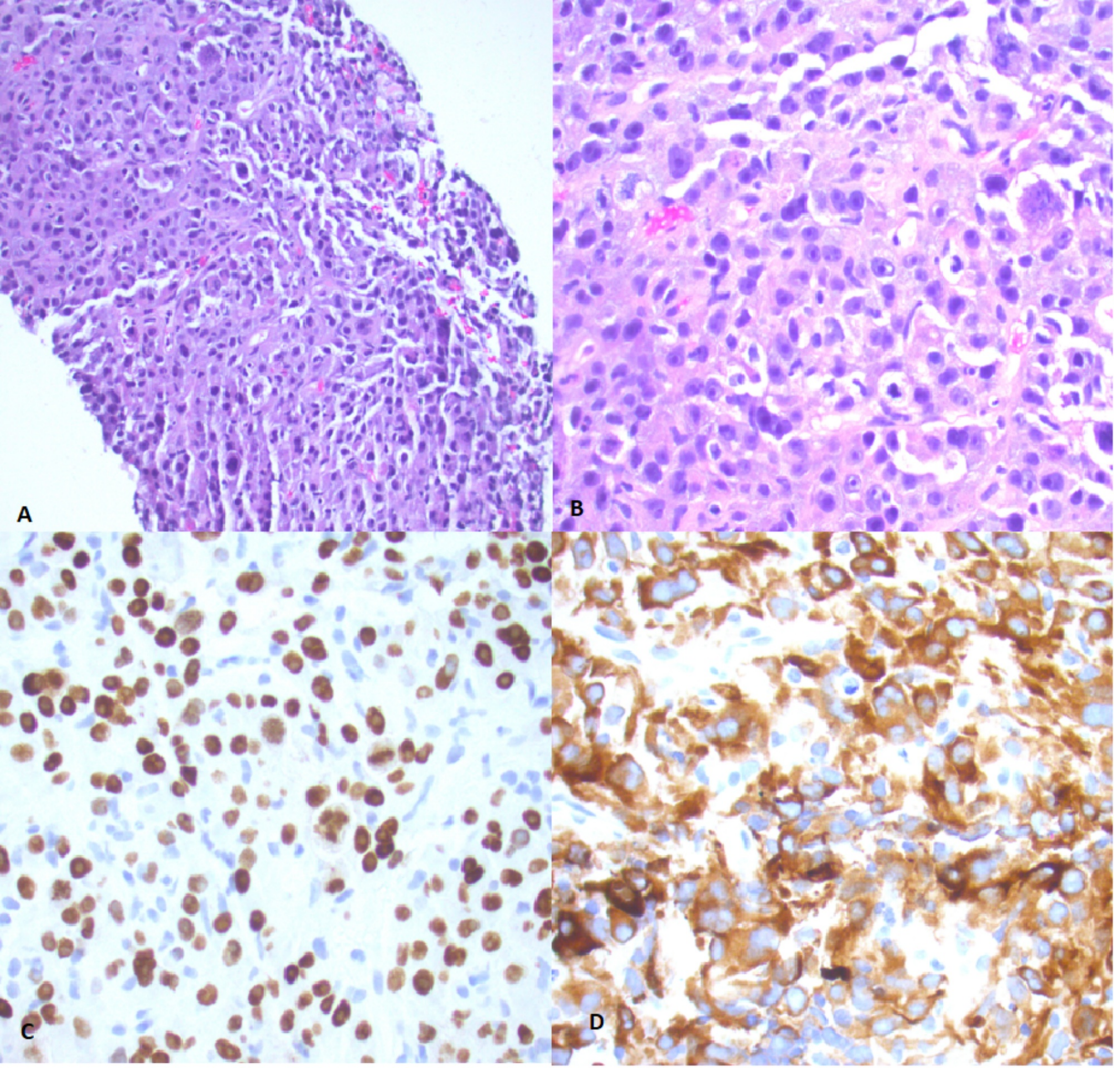
Mediastinal biopsy tissue A: Gross tissue sample demonstrating poorly-differentiated non-small cell lung carcinoma (NSCLC); B: This is a close-up of the cellular composition showing pleomorphic, hyperchromatic nuclei, variably prominent nucleoli, and small to moderate amounts of cytoplasm. These are all classic hallmarks of NSCLC; C: Positive TTF-1 staining; D: Positive cyto-keratin AE1/AE3

The patient underwent a mediastinal biopsy and was diagnosed with metastatic poorly differentiated non-small cell adenocarcinoma (Figure 3). She later underwent a bronchoscopy with tracheal stent placement. Her right arm pain, numbness, and tingling eventually resolved after treatment with systemic steroids. She is now undergoing systemic chemoimmunotherapy with carboplatin/pemetrexed although she has recurrent admissions to the hospital for chronic malignant pleural effusions and chest pain, requiring tunneled pleural catheter placement. 

## Discussion

Literature about the clinical characteristics, pathophysiology, and time trends of young patients with lung cancer is very limited, having only a 1.37% incidence rate in ages of 18 to 35 [2]. However, one study called the Surveillance, Epidemiology and End Results (SEER) database created in 2010 gives us some insight regarding the diagnosis of NSCLC in young adults. Younger patients were more likely to have adenocarcinoma, while older patients were more likely to have squamous cell carcinoma [3]. Generally, about 70% of patients diagnosed with lung cancer present with advanced disease [4]. More specifically, younger patients make up about 0.6% of NSCLC diagnoses and were more likely to present with advanced-stage NSCLC. When race was taken into account, female African-Americans and Pacific Islanders were the most likely to present with advanced features, similar to our patient [3].

One study, looking at features of NSCLC in young patients, noted that non-smokers were less likely to have upper lobe cancers, which our patient presented with on imaging [5]. It has been hypothesized that non-smokers who have lung cancer have mechanisms of disease that are different from patients with a smoking history. Researchers have proposed risk factors for the development of NSCLC in never-smokers are weak to modest at best and require more investigation [6]. One suggested mechanism of carcinogenesis is the upregulation of estrogen-alpha and -beta receptors in cancer cells, which may explain why the incidence of NSCLC is higher in non-smoking women [7]. Another mechanism to consider is the epidermal growth factor receptor (EGFR) mutation pathway that appears to be affected in non-smoking patients who develop adenocarcinoma [3]. Another risk factor that has been suggested is that women are likely to be more exposed to cooking fumes, which may be associated with a higher risk of lung cancer [6].

NSCLC is commonly treated with surgery and adjuvant therapy from stages I-IIIA. For stage IV patients, like our patient, the first-line therapy is usually platinum plus pemetrexed, especially if a patient has confirmed adenocarcinoma. Though this is a recommended first-line treatment, other chemotherapeutics were found to be equally efficacious. The median lifespan after starting treatment is eight to ten months [7]. Other studies have shown statistical significance with treatment of lung cancer with platinum-based regimens among women [8].

Our young African-American female patient, presented in this case report, had no modifiable risk factors to develop NSCLC. On presentation, her only symptom was right arm pain without any respiratory complaints or symptoms. Despite this benign appearing presentation, she was found to have severe tracheal stenosis due to a large lung mass, which do not commonly present together in lung cancer. One case report presents a case of a gentleman who had right shoulder pain and was found to have lung cancer that presented as metastatic disease to the scapula and spine. He did not have any tracheal deviation as a result [9]. Another case report presents a case in which a woman desaturated during bronchoscopy secondary to tracheal stenosis secondary to lung cancer; however, she did not report any pain symptoms [10]. In terms of prognosis, young non-smokers with regional disease had better survival outcomes [3].

## Conclusions

The low prevalence of non-small cell lung cancer in the young patient population combined with the differences in clinical presentation described in this case makes this a very imperative topic for the healthcare team to be aware of to prevent delayed diagnosis and treatment as well as to improve patient outcomes. Additionally, a better understanding of other risk factors, besides age, smoking, exposures to chemicals, etc., is needed in order to develop targeted therapeutics.
